# A Memoir of Samuel George Morton, M. D.

**Published:** 1852-02

**Authors:** 


*A Memoir of Samuel George Morton, M. D.* By CHARLES D. MEIGS, M. D., *Read before the Academy of Natural Sciences, November 6th*, 1851.

This elegant biography will be read with the deepest interest by the wide circle of the friends and admirers of Dr. Morton. It is in Dr. Meigs' happiest style, abounding in the genial earnestness and graceful bonhommie, which are so well known as his characteristics, and no less distinguished by erudition, tact, and right feeling. We should take the occasion to extract at length from it, but for the extended notice of Dr. Morton, which appeared in the number of this Journal for June last.

